# An Update on the Treatment of Papillary Renal Cell Carcinoma

**DOI:** 10.3390/cancers15030565

**Published:** 2023-01-17

**Authors:** Neal S. Chawla, Nicolas Sayegh, Sweta Prajapati, Elyse Chan, Sumanta K. Pal, Alexander Chehrazi-Raffle

**Affiliations:** 1 Department of Medical Oncology & Experimental Therapeutics, City of Hope Comprehensive Cancer Center, Duarte, CA 91010, USA; 2 Division of Oncology, Department of Internal Medicine, Huntsman Cancer Institute, University of Utah, Salt Lake City, UT 84112, USA

**Keywords:** papillary, renal cell carcinoma

## Abstract

**Simple Summary:**

Papillary renal cell carcinoma is the second most common type of kidney cancer, after clear cell kidney cancer. Given that it is relatively rare, studying this disease has been quite a challenge. New treatments and techniques for studying papillary kidney cancer have led to some meaningful improvements in therapy for this disease. In this review article, we summarize some of the historical studies in this space, and look ahead to three upcoming trials in papillary renal cell carcinoma.

**Abstract:**

Papillary renal cell carcinoma (pRCC) is the second-most common subtype of kidney cancer following clear cell renal cell carcinoma (ccRCC), representing 15% of kidney cancers. Despite advances in therapy, including combination strategies with targeted therapies and immune checkpoint inhibitors, progress has lagged behind that of ccRCC. This is in part due to the heterogenous nature of the various subtypes of pRCC. More recently, investigators have turned efforts towards histology and biology-based trials. In this review, we outline some of the distinct biological characteristics of pRCC and discuss the most impactful clinical trials to date. Finally, we look ahead to several highly anticipated ongoing trials in pRCC.

## 1. Introduction

There will be an estimated 79,000 cases of renal cell carcinoma (RCC) in 2022, of which non-clear renal cell carcinoma (nccRCC) will represent approximately 25–30% [1,2]. The most recent global statistics indicate that there were over 400,000 cases of RCC in 2020 [3]. nccRCC is comprised of several subtypes, including papillary (pRCC), chromophobe, collecting duct, renal medullary, translocation, and unclassified. pRCC represents 15% of kidney cancers and the most common type of nccRCC [4].

pRCC is diagnosed most commonly between the ages of 50–70 and occurs more often in men [5]. These tumors are often characterized radiologically by calcification and are frequently multifocal in nature [6,7]. Although retrospective and limited in sample size, Dudani and colleagues’ analysis of the International Metastatic RCC Database Consortium (IMDC) suggested that metastatic pRCC is associated with worse survival compared to metastatic clear cell RCC (ccRCC), regardless of the site of metastasis [8]. pRCC has a threefold-higher incidence among Black people than White people [5]. Additionally, Asian Americans have been found to have an increased risk of pRCC based on data from a regional database with nearly 10,000 patients [9].

In metastatic renal cell carcinoma (mRCC), randomized phase III trials have predominantly focused on ccRCC. Consequently, the treatment paradigm of advanced nccRCC has historically been predicated on retrospective data, phase II trials, and subgroup analyses of phase III trials. In this review, we outline the biological characteristics of pRCC with a particular focus on treatment in the advanced/metastatic setting.

## 2. Biological Subtypes

Previously, there were two commonly recognized histological patterns of pRCC. However, emerging data has identified several additional subtypes that are biologically distinct [10]. As many of the studies discussed herein adopt the traditional terminology, we retain this while reviewing the data pertaining to these trials.

Type 1 pRCC is characterized by cells in a single layer with oval nuclei and with scant basophilic cytoplasm [11]. On immunohistochemistry, CK7, MUC1, and vimentin expressions are common in type 1 pRCC [12]. Type 2 pRCC is typically made up of large pseudostratified, eosinophilic cells with prominent nucleoli and round nuclei [11,13]. CK20 and E-cadherin expressions occur more often in type 2 pRCC [12]. Molecular studies suggest that type 2 pRCC may, in fact, be comprised of several different entities with distinct molecular profiles [11]. Several subtypes that were previously considered type 2 are now distinct entities in the 2016 WHO classification, including hereditary leiomyomatosis and renal cell cancer (HLRCC) and translocation renal cell carcinoma [13].

More recently, next generation sequencing (NGS) has elucidated three prominent genomic clusters of pRCC. Comprehensive molecular profiling of 161 tissue samples from The Cancer Genome Atlas (TCGA) database showed that type 1 pRCC was largely driven by mesenchymal-epithelial transition factor (MET) pathway alterations [11]. Type 1 pRCC tended to be lower-grade and exhibited higher rates of chromosomal gains in 7p and 17p. Type 2 pRCC appeared to have two distinct subtypes: one with less copy number alterations and the other with multiple chromosome losses, in particular chromosome 9p. Although the cluster with multiple chromosome losses portended worse survival than its counterparts, it is important to note that these specimens were largely from patients with non-metastatic disease (97%) [11].

In an effort to obtain data that may be more representative of a real-world population, NGS was conducted on samples from 169 patients with pRCC, 61% of which had metastatic disease [14]. *MET* alterations were seen in 33 of 39 (85%) patients with type 1 pRCC and 7 of 108 (6%) patients with type 2 pRCC. However, a study of 220 pRCC tumor samples by the French RCC Network also highlighted the role of *MET* copy number alterations, which were detected at a rate of 81% and 46% for type 1 and type 2 pRCC, respectively [15]. Given the prevalence of MET signaling alterations in pRCC, numerous studies have explored agents that inhibit this receptor; however, as discussed in this review, these efforts have been fraught with challenges in deriving clinically meaningful benefit.

Additionally, pRCC tumors have been shown to originate from multiple different cell types. Single-cell assays for transposase-accessible chromatin-sequencing (scATAC-seq) data suggested that some pRCC tumors can originate from collecting duct (CD) cells, besides classically originating from proximal tubule (PT) cells [16]. While PT origin was associated with an enrichment in the NOTCH and mTOR pathways, pRCC tumors with CD origin manifested a higher expression of inflammation pathways and interferon signaling. Moreover, these tumors were correlated with a higher risk of progression to an advanced stage.

## 3. Targeted Therapies

### 3.1. mTOR and VEGF Inhibitors

In the cytokine era, the phase III ARCC trial compared interferon-α (IFN) with a mammalian target of rapamycin (mTOR)-inhibitor temsirolimus in patients with previously untreated mRCC [17]. mTOR inhibitors bind with intracellular protein FKBP-12, resulting in disruption of the mTOR pathway, which is frequently altered in RCC [18,19]. Based on the overall survival (OS) benefit seen with single-agent temsirolimus versus IFN in a pivotal phase III trial, there was subsequent interest in the potential of this agent in nccRCC [20]. With 206 patients in each treatment arm, there were 30 (15%) and 25 (12%) patients with pRCC in the IFN and temsirolimus cohorts, respectively. Median progression-free survival (PFS) for pRCC patients was 1.8 months with IFN versus 3.8 months with temsirolimus (95% CI, 0.28–0.83). Median OS in the pRCC cohort was 5.7 months with IFN, compared to 10.9 months for patients treated with temsirolimus (95% CI, 0.27–0.94). Although limited by the small sample size, these findings suggested the superiority of temsirolimus over IFN in pRCC.

The RAPTOR trial, a single-arm phase II trial of everolimus in previously untreated pRCC (32% with type 1 and 64% with type 2), enrolled a total of 88 patients in its intention-to-treat (ITT) cohort [21]. Results were notable for an OS of 21.4 months (95% CI, 15.4–28.4) despite a very low response rate of 1%. The substantially longer OS with everolimus in the RAPTOR trial, compared to temsirolimus in the ARCC trial, may in part be attributable to the high number of patients (65%) in the RAPTOR trial that exhibited stable disease (SD) as a best response. Furthermore, as noted by the authors, a significant number of patients in RAPTOR continued therapy with everolimus in spite of progressive disease, which likely contributed to the high OS in this study. The results of ARCC and RAPTOR established mTOR inhibition as a frontline strategy in pRCC.

With the introduction of vascular endothelial growth factor (VEGF)-pathway tyrosine kinase inhibitors, two early studies signaled that these agents held promise in the non-clear cell setting. Tissue-based- and mRNA-expression studies indicated that pRCC exhibits substantial expression of VEGF and VEGF receptors [22,23]. Furthermore, MET mutations in type 1 pRCC were shown to lead to VEGF transcription via overaccumulation of hypoxia-inducible factor, augmenting the biological rationale of testing these agents in pRCC [24]. A single-arm phase II study of sunitinib in nccRCC included 22 (71%) patients with pRCC, in whom the response rate was 36% [25]. In the overall cohort, median PFS was 6.4 months (95% CI, 4.2–8.6) and median OS was 25.6 months (95% CI, 8.4–42.9). In addition, sunitinib was evaluated in the SUPAP trial, which enrolled 61 pRCC patients (15 patients with type 1 and 46 patients with type 2 pRCC) [26]. In this single-arm phase II trial, median PFS was 6.6 months (95% CI, 2.8–14.8) and 5.5 months (95% CI, 3.8–7.1), and OS was 17.8 months (95% CI, 5.7–26.1) and 12.4 months (95% CI, 8.2–14.3), in type 1 and type 2 pRCC, respectively. These studies both affirmed that VEGF inhibition is an effective therapeutic strategy in pRCC.

Following these encouraging results, investigators prospectively evaluated VEGF inhibitors versus mTOR inhibitors in two multicenter randomized phase II trials that enrolled predominantly nccRCC patients: ESPN and ASPEN. A total of 108 patients were enrolled in the ESPN trial, which randomized patients to either first-line sunitinib or everolimus with crossover at progression. Overall median PFS on first-line therapy with sunitinib was 6.1 months (95% CI, 4.2–9.4), compared to 4.1 months (95% CI 2.7–10.5) with first-line everolimus (*p* = 0.60) [27]. A total of 27 (25%) patients with pRCC were included, with a median PFS of 5.7 months, (95% CI, 1.4–19.8) with first-line sunitinib, compared to 4.1 months (95% CI, 1.5–7.4) with upfront everolimus. Median OS was 16.6 months (95% CI, 5.9-NA) vs. 14.9 months (95% CI, 7.1–22.7) in patients who received front-line sunitinib and everolimus, respectively. Among the pRCC cohort, 1/33 (3%) patients had a partial response (PR) with first-line sunitinib, with 1/23 (4%) patients achieving a PR with second-line everolimus. No objective responses were seen with upfront everolimus in patients with pRCC.

In the ASPEN trial, 108 patients with either pRCC, chromophobe RCC, or unclassified RCC were randomized to receive either sunitinib or everolimus [28]. With a primary endpoint of PFS in the intention-to-treat cohort, sunitinib demonstrated a significantly improved PFS of 8.3 vs. 5.6 months with everolimus (*p* = 0.16, HR 1.41; 80% CI, 1.03–1.92). Overall survival (OS) was not significantly different between the two trial arms. Among 70 (65%) patients with pRCC, median PFS was 8.1 vs. 5.5 months (HR 1.6, 80% CI, 1.1–2.3) with sunitinib and everolimus, respectively [28].

Taken together, the ESPN and ASPEN trials largely supported the use of sunitinib in pRCC, although this was limited due to a small sample size. A meta-analysis that included these studies showed that sunitinib demonstrated a superior PFS compared to everolimus in nccRCC (*p* < 0.00001, HR 0.67 (0.56–0.80)), although there was no difference seen in OS [29]. While the heterogenous populations and the limited sample size makes it difficult to draw strong conclusions, sunitinib appeared to have more activity than everolimus in these trials. These studies paved the way for future studies with this class of therapy in pRCC.

Axitinib, a more specific and potent VEGF inhibitor, was assessed in the AXIPAP trial, a multicenter phase II single-arm study that included 44 patients with pRCC [30]. In this previously untreated cohort, 13 patients had type 1 pRCC, 30 had type 2 pRCC, and 1 had unspecified papillary histology. With a median follow-up of 32 months, the median PFS was 6.6 months in patients with type 1 disease (95% CI, 5.5–9.2) and 6.2 months in type 2 disease (95% CI, 5.4–9.2). The median PFS at 24 weeks, which was the primary endpoint, was 45.2% (95% CI 32.6%-NR) in the overall cohort and 46.2% (95% CI, 23.4-NR) and 42.9% (95% CI, 27.5-NR) in type 1 and type 2 pRCC subgroups, respectively. The ORR was 28.6% (95% CI, 15.7–44.6), and higher response rates of 35.7% were seen in the type 2 subgroup compared to 7.7% in type 1 patients. This suggests more reliance on the VEGF pathway in type 2 pRCC compared to type 1 pRCC.

Given the activity of single-agent targeted therapy in nccRCC, attention then turned toward the efficacy of doublet therapy. Based on the activity of lenvatinib (a multikinase inhibitor with predominant anti-VEGF activity) with everolimus in ccRCC following one line of VEGF-directed therapy, Hutson et al. evaluated the regimen in nccRCC [31,32]. In this phase II single-arm study of previously untreated unresectable advanced or metastatic nccRCC, patients received lenvatinib/everolimus with a primary endpoint of safety and tolerability. Out of 31 patients, 20 (65%) had papillary histology. Median PFS in the pRCC group was 9.2 months (95% CI, 3.5-NE), and median OS was 11.7 months (95% CI, 8.1-NE). Overall, the results of this trial were promising for a combination approach in pRCC.

### 3.2. MET Inhibitors

In light of the central role of the MET pathway in type 1 pRCC (and to a lesser degree type 2 pRCC), researchers sought to interrogate MET inhibitors in pRCC. Foretinib, one of the first MET inhibitors, was assessed in a phase II trial at two dosage levels with a primary endpoint of ORR [33]. In total, 74 patients were enrolled, with an ORR of 13.5% (95% CI, 6.7–23.5%) and a median PFS of 9.3 months (95% CI, 6.9–12.9). Notably, 5 of 10 patients (50%) with germline *MET* mutations had a response, compared to 5/57 (9%) patients that did not harbor these mutations, bolstering the utility of targeting this pathway.

SAVOIR was a randomized phase III trial that employed a biomarker-driven approach to MET inhibition in pRCC. Criteria for enrollment included the presence of chromosome 7 gain, amplification of *MET*, mutation of the *MET* kinase domain, or alteration of hepatocyte growth factor [34]. With a total accrual of 60 patients, the study was terminated early. However, it should be noted that savolitinib showed a numerically superior response rate to sunitinib, 27% (95% CI, 13.3–45.5) vs. 7% (95% CI, 0.9–24.3), respectively [34].

Several other targeted drugs with anti-MET pathway activity were considered potential candidates in the therapy of pRCC. The PAPMET trial enrolled pRCC patients in one of the four drugs with anti-MET activity: sunitinib, cabozantinib, crizotinib, and savolitinib [35]. In this multicenter study, 147 patients (with ≤1 line of therapy that did not include a VEGF or MET inhibitor) received one of the four aforementioned agents, with a primary endpoint of PFS. Based on prespecified futility analysis, randomization of the crizotinib and savolitinib cohorts stopped at 29 and 28 patients, respectively. Cabozantinib, which is a multikinase inhibitor targeting VEGF, MET, and AXL, demonstrated a superior median PFS of 9.0 months, compared to sunitinib with 5.6 months (95% CI, 0.37–0.97, *p* = 0.019). Cabozantinib also had a 23% response rate, compared to 4% for sunitinib (*p* = 0.010). Savolitinib and crizotinib fared worse than sunitinib in terms of PFS. These data firmly established cabozantinib as the preferred frontline therapy for pRCC.

## 4. Immunotherapy

The advent of immune checkpoint inhibitors (ICIs)—antibodies that target either programmed cell death-1 (PD-1), its ligand (PD-L1), or the cytotoxic T-lymphocyte-associated antigen (CTLA-4)—transformed the treatment landscape for advanced/metastatic ccRCC [36,37]. Although there are no phase III trials of ICIs in nccRCC to date, several phase II trials have provided valuable information regarding these agents’ biologic activity in this setting.

KEYNOTE-427 was a multicenter, single-arm, phase II study in which first-line pembrolizumab (PD-1 inhibitor) monotherapy was investigated in 165 patients with non-clear cell histology [38]. Among those, 71.5% had confirmed pRCC. In this subgroup, ORR was 28.8% (95% CI, 20.8–37.9). Disease control rate, median PFS, and median OS were 47.5% (95% CI, 38.2–56.9), 5.5 months (95% CI, 3.9–6.9), and 31.5 months (95% CI, 25.5-NR), respectively. A complete response (CR) rate of 5.9% was observed amongst the papillary cohort.

In a multicenter retrospective study of 41 patients with treatment-refractory advanced/metastatic nccRCC, the anti-PD-1 agent nivolumab demonstrated a median PFS of 3.5 months (95% CI, 1.9–5.0) [39]. Of the 16 (39%) patients with pRCC, 2 patients achieved a PR and 3 patients had stable disease (SD). CheckMate-374 was a prospective, multicenter, single-arm study that included 44 patients with nccRCC who were treated with nivolumab, with a primary endpoint of safety [40]. The ORR was 13.6% (95% CI, 5.2–27.4), including 1 CR (2.3%) and 5 PRs (11.4%). Though the median PFS was 2.2 months (95% CI, 1.8–5.4), there was an impressive median OS of 16.3 months (95% CI, 9.2-NE). A total of 24 (55%) pRCC patients were included, with 5 (11.4%) of these patients having a PR and 9 (20%) with SD as a best response. PD-L1 status did not appear to have a significant impact on the efficacy of nivolumab in these patients.

Although pembrolizumab appears to have outperformed nivolumab, it is notable that the former enrolled only untreated patients, whereas the latter included 34% of patients who had received prior therapy. Furthermore, there was a greater preponderance of patients with papillary histology in KEYNOTE-427, which likely contributed to an improved response compared to that of CheckMate-374. Notwithstanding these differences, KEYNOTE-427 and CheckMate-374 established the role of single-agent immunotherapy in nccRCC.

Checkmate-214 was a randomized phase III trial that established the role of doublet immune checkpoint inhibition (CTLA-4 blockade along with PD-1 inhibition) in ccRCC, which had superior efficacy over sunitinib in the intermediate/poor IMDC risk group [36,41]. These findings spurred interest in this combination in nccRCC. CheckMate-920 was a single-arm phase III/IV trial that included 52 patients with previously untreated advanced/metastatic nccRCC [42]. The response rate was 19.6%, with a median PFS and OS of 3.7 months (95% CI, 2.7–4.6) and 21.2 months (95% CI, 16.6-NE), respectively. Patients with pRCC made up 34.6% of the cohort, of whom five patients had a response (1 CR, 4 PR). These findings were further bolstered by several studies recently reporting retrospective and systematic review data, which, once again, showed the potential for response to immune checkpoint inhibition in advanced/metastatic pRCC [43,44,45].

## 5. Combination Therapy

While the therapeutic strategy of using single-agent VEGF-TKIs, mTOR inhibition, MET inhibitors, and ICIs each demonstrated some activity in pRCC, these paled in comparison to that of the findings in ccRCC. Interest in the potential synergy of these combinations in pRCC was in part due to the impressive response rates of combination cabozantinib/nivolumab in CheckMate 9ER in ccRCC [46]. A single-arm phase II trial is investigating the combination in patients with advanced nccRCC, allowing a maximum of one line of prior non-ICI therapy [47]. Cohort 1 of the study, with a total of 40 patients, included 32 (80%) patients with pRCC, 6 (15%) patients with unclassified-without-papillary features, and 2 (5%) patients with tRCC, demonstrating an impressive 48% (95% CI, 31.5–63.9) ORR. Similarly, COSMIC-021 evaluated atezolizumab, an anti-PD-L1 monoclonal antibody, plus cabozantinib across 102 patients. Among the 15 patients in this cohort with pRCC, 47% had an objective response (Table 1).

In an effort to leverage the MET pathway along with an ICI, CALYPSO was a single-arm phase II study that treated pRCC patients with the combination of durvalumab (a PD-L1 inhibitor) plus savolitinib [51]. Previous lines of therapy were allowed. ORR was 27% among the 42 patients that were enrolled. After a median follow-up of 8.9 months, median PFS was 3.3 months in the overall cohort and 12 months in the subgroup of previously untreated patients. Molecular analysis showed that 14 of the enrolled patients had MET-driven disease, in whom the response rate was 57% [52].

Other combination strategies in this setting include bevacizumab (a monoclonal antibody targeting VEGF) in combination with atezolizumab, which was assessed in a multicenter phase II study [53]. With a total of 60 patients enrolled, papillary histology was identified in 12 (20%) patients with a response rate of 25%. Similarly, KEYNOTE-B61 was a phase II single-arm trial of the combination of pembrolizumab and lenvatinib, which demonstrated considerable activity in pRCC, with an ORR of 52.9% (95% CI, 38.5–67.1) across 51 patients [50].

Given the promising potential for these treatment modalities, there is an urgent need for predictive biomarkers. A study of PD-L1 expression on formalin-fixed paraffin-embedded tumor samples from 101 patients with nccRCC was reported in 2014, showing low tumor cell expression and modest tumor-infiltrating mononuclear cell expression of PD-L1 [54]. Tumor mutational burden (TMB) has not been an effective biomarker in pRCC, as it tends to be relatively low, with one study showing a median TMB of 2.7 Mb in patients with pRCC in both the type 1 and type 2 diseases [14]. Whole exome sequencing was performed on tumor samples in the aforementioned study of cabozantinib/nivolumab showed that 10 of 12 patients who harbored *NF2* or *FH* mutations exhibited an objective response to the combination [47]. Of note, only 1 of 6 patients with a *SETD2* had a response. Larger studies are needed to validate these molecular findings as potential markers of responses to TKI/ICI combinations.

## 6. Ongoing Trials in pRCC

**Figure 1:**
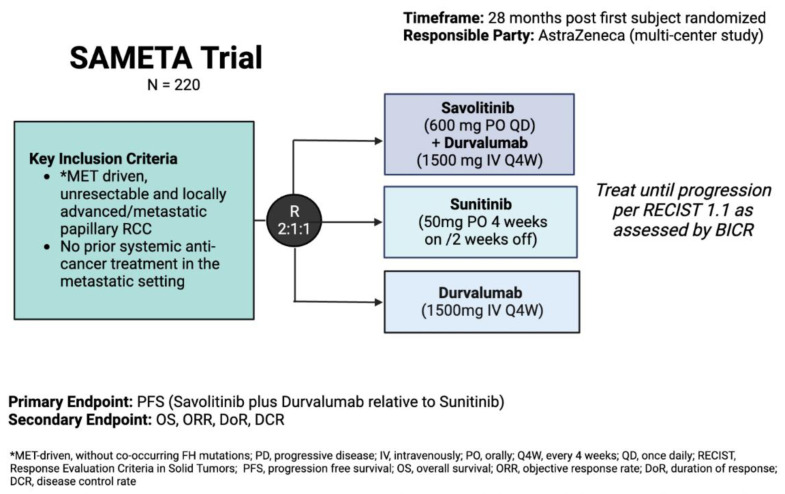
SAMETA trial schema (NCT05043090).

At present, there are three highly anticipated prospective trials of VEGF/ICI, doublet-ICI, and MET-inhibitor/ICI combination strategies in pRCC. As a follow-up to the impressive results of cabozantinib in the PAPMET trial, PAPMET 2 (NCT02761057) is a randomized phase II study of cabozantinib with or without atezolizumab in pRCC. This trial has a target enrollment of 200 patients who have had < 1 line of prior therapy with a primary endpoint of PFS (Figure 1).

Although there appears to be promising evidence for doublet-ICI based on single-arm and retrospective data in pRCC, prospective randomized data comparing the combination to standard-of-care regimens is needed. SUNIFORCAST (NCT03075423) is an ongoing phase II multicenter study comparing the combination of ipilimumab/nivolumab to standard-of-care physician’s-choice regimens in patients with advanced nccRCC [55]. Target accrual is 306 patients, with a primary endpoint of 12-month OS. Secondary endpoints include the 6-month and 18-month OS, median OS, PFS, ORR, and quality-of-life metrics. This trial is expected to include a considerable number of patients with advanced/metastatic pRCC.

**Figure 2:**
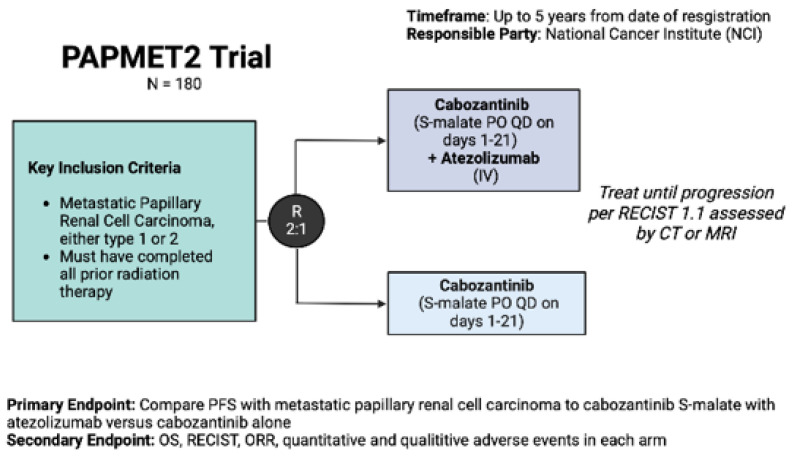
PAPMET 2 trial (NCT05411081).

Building off the success of CALYPSO, SAMETA (NCT05043090) is a randomized phase III, three-arm trial comparing the combination of savolitinib and durvalumab against monotherapy with either sunitinib or durvalumab in patients with advanced/metastatic pRCC. Target enrollment is 220 participants, with a primary endpoint of PFS of the combination versus sunitinib (Figure 2).

## 7. Conclusions

pRCC is the second-most common histologic subtype of kidney cancer and has benefited from the therapeutic advances of its more-prevalent clear-cell counterpart. Historically, early phase or subgroup analyses spurred the development of nccRCC- or pRCC-specific phase II trials from which treatment algorithms were largely devised. However, there remains a paucity of pRCC-specific randomized phase III trials. The current standard for metastatic pRCC should theoretically be cabozantinib, but guidelines remain vague around whether this or sunitinib are the most appropriate options. Thus, ongoing studies such as SAMETA, which is structured as a phase III registration trial, will be helpful. Other phase III studies in this space are eagerly anticipated.

On the horizon, there are several clinical trials that seek to leverage the potential synergies of VEGF/ICI, doublet-ICI, and MET-inhibitor/ICI combinations. As we chart a path forward in pRCC, it will be of utmost importance to design large-scale clinical trials that adequately evaluate and validate the nuanced biology and demography of this disease state.

## Figures and Tables

**Table 1:** Notable trials in papillary renal cell carcinoma.

Trial	Inclusion Criteria	Number of Patients (Papillary)	Experimental Arm	Control Arm	Results
**RAPTOR** **(NCT00688753)**	• Locally advanced or metastatic type 1 or type 2 papillary RCC. • No previous systemic treatment	88 (88)	First-line everolimus 10 mg orally daily	N/A	OS (median): 21.4 months (95% CI 15.4–28.4) PFS (median): 4.1 months (95% CI 3.6–5.5)
**SUPAP** [26] **(NCT00541008)**	• Locally advanced or metastatic type 1 or type 2 papillary RCC. • No previous systemic treatment	61 (61)	Sunitinib 50 mg orally daily every 4 weeks followed by 2 weeks without treatment	N/A	OS (median): 17.8 months (95% CI, 5.7–26.1) (type 1 pRCC) and 12.4 months (95% CI, 8.2–14.3) (type 2 pRCC) PFS (median): 6.6 months (95% CI, 2.8–14.8) (type 1 pRCC) and 5.5 months (95% CI, 3.8–7.1) (type 2 pRCC)
**ESPN** [27] **(NCT01185366)**	• Locally advanced or metastatic non-clear cell RCC. • No previous systemic treatment	108 (27)	First-line everolimus 10 mg orally daily (Crossover at progression allowed)	First-line sunitinib 50 mg orally every day (4 weeks, 6-week cycles) (Crossover at progression allowed)	OS (median): 14.9 months (95% CI, 7.1–22) vs. 16.6 (95% CI, 5.9-NA) ^A^ PFS (median): 4.1 months (95% CI, 1.5–7.4) vs. 5.7 months (95% CI, 1.4–19.8) ^A^ ORR: 4% vs. 3% (first-line) ^A^
**ASPEN** [28] **(NCT01108445)**	• Locally advanced or metastatic non-clear cell RCC. • No previous systemic treatment	108 (70)	Everolimus 10 mg orally daily	Sunitinib 50 mg orally every day (4 weeks, 6-week cycles)	PFS (median): 5.5 vs. 8.1 months (HR 1.6; 80% CI, 1.1–2.3) ^A^
**AXIPAP** [30] **(NCT02489695)**	• Locally advanced or metastatic papillary RCC. • No previous treatment with a tyrosine kinase inhibitor in the adjuvant setting	44 (44)	Axitinib 10 mg orally twice daily	N/A	24-week progression-free rate: 45.2% (95% CI, 32.6–+∞) PFS (median): 6.6 months (95% CI, 5.5–9.2)
**Hutson et al.** **(NCT02915783)**	• Locally advanced or metastatic non-clear cell RCC. • No previous systemic treatment	31 (20)	Lenvatinib 18 mg plus everolimus 5 mg orally once daily	N/A	PFS (median): 9.2 months (95% CI, 3.5-NE) ^A^ OS (median): 11.7 months (95% CI, 8.1-NE) ^A^
**Choueiri et al.** **(NCT00726323)**	• Locally advanced or metastatic papillary RCC. • Up to one previous line of therapy is allowed	74 (74)	Foretinib 240 mg orally daily (with days 1 to 5 every 14 days for cohort A) or 80 mg orally daily (cohort B)	N/A	ORR: 13.5% (95% CI, 6.7–23.5) PFS (median): 9.3 months (95% CI, 6.9–12.9)
**SAVOIR** [34] **(NCT03091192)**	• Locally advanced or metastatic papillary RCC • MET-driven papillary RCC without co-occurring FH or VHL mutations from tumoral next-generation sequencing • Previous systemic therapy allowed (except sunitinib or another MET inhibitor)	60 (60)	Savolitinib 600 mg orally daily	Sunitinib 50 mg orally every day (4 weeks, 6-week cycles)	PFS (median): 7.0 vs. 5.6 months (HR 0.71; 95% CI, 0.37–1.36; *p* = 0.31) ORR: 27% (95% CI, 13.3–45.5) vs. 7% (95% CI, 0.9–24.3)
**PAPMET** [35] **(NCT02761057)**	• Locally advanced or metastatic papillary RCC. • Up to one previous line of therapy is allowed (except for another VEGF-TKI)	147 (147)	Cabozantinib 60 mg orally daily, crizotinib 250 mg orally daily, savolitinib 600 mg orally daily, or sunitinib 50 mg orally daily (with 4 weeks on and 2 weeks off)	N/A	Cabozantinib vs. crizotinib vs. savolitinib vs. sunitinib PFS (median): 9 vs. 2.8 vs. 3 vs. 5.6 months OS (median): 20 vs. 19.9 vs. 11.7 vs. 16.4 months
**KEYNOTE-427** [38] **(NCT02853344)**	• Locally advanced or metastatic non-clear cell RCC (cohort B). • No previous systemic treatment (except neoadjuvant/adjuvant completed > 12 months prior to allocation)	165 (118)	Pembrolizumab 200 mg intravenously every 3 weeks	N/A	ORR: 28.8% (95% CI, 13.3–45.5) ^A^ DCR: 47.5% (95% CI, 38.2–56.9) ^A^ PFS (median): 5.5 months (95% CI, 3.9–6.9) OS (median): 31.5 months (95% CI, 25.5-NR) ^A^
**CheckMate 374** [40] **(NCT02596035)**	• Locally advanced or metastatic non-clear cell RCC • Up to three lines of previous systemic therapy allowed	44 (24)	Nivolumab 240 mg intravenously every 2 weeks	N/A	ORR: 13.6% (95% CI, 5.2–27.4) PFS (median): 2.2 months (95% CI, 1.8–5.4) OS (median): 16.3 months (95% CI, 9.2-NE)
**CheckMate 920** [48] **(NCT02982954)**	• Locally Advanced or metastatic non-clear cell RCC • No previous systemic treatment allowed	52 (22)	Nivolumab (3 mg/kg) plus ipilimumab (1 mg/kg) every 3 weeks (four doses); then nivolumab 3 mg/kg every 2 weeks	N/A	PFS (median): 3.7 months (95% CI, 2.7–4.6) OS (median): 21.2 months (95% CI, 16.6-NE)
**Lee et al.** **(NCT03635892)**	• Locally advanced or metastatic non-clear cell RCC • No previous systemic treatment	47 (40)	Nivolumab 240 mg intravenously every 2 weeks plus cabozantinib 40 mg orally daily	N/A	ORR: 48% (95% CI, 31.5–63.9)
**COSMIC 021** [49] **(NCT03170960)**	• Locally advanced or metastatic solid tumor. • No previous systemic treatment with cabozantinib or immune checkpoint inhibitors	102 (15)	Cabozantinib 40 mg orally daily plus atezolizumab 1200 mg intravenously every 3 weeks	N/A	ORR: 19.6% (95% CI, 9.4–33.9) PFS (median): 3.7 months (95% CI, 2.7–4.6) OS (median): 21.2 months (95% CI, 16.6-NE)
**McGregor et al.** **(NCT02724878)**	• Locally advanced or metastatic non-clear cell RCC	60 (12)	Atezolizumab 1200 mg and bevacizumab 15 mg/kg intravenously every 3 weeks	N/A	ORR: 47.5% (95% CI, 31.5–63.9) PFS (median): 12.5 months (95% CI, 6.3–16.4) OS (median): 28 months (95% CI, 16.3-NE)
**KEYNOTE-B61** [50] **(NCT04704219)**	• Locally advanced or metastatic non-clear cell RCC • No previous systemic treatment allowed	82 (51)	Lenvatinib 20 mg orally daily plus pembrolizumab 200 mg intravenously every 3 weeks	N/A	ORR: 47%

^A^ Results pertain to the papillary cohort only. Abbreviations: RCC, renal cell carcinoma; OS, overall survival; PFS, progression free survival; ORR, objective response rate; DCR, disease control rate.
